# Explaining Immigrants' Worries About Ethnic Harassment: Germany, 1986–2004

**DOI:** 10.3389/fsoc.2020.538878

**Published:** 2020-10-09

**Authors:** Christoph Spörlein, Elmar Schlueter

**Affiliations:** ^1^Institute of Social Sciences, Heinrich Heine University, Duesseldorf, Germany; ^2^Institute for Sociology, Justus Liebig University, Giessen, Germany

**Keywords:** interethnic relations and conflicts, prejudice, discrimination, mass media, multlilevel modeling, immigrants

## Abstract

What factors shape immigrants' worries about becoming targets of ethnic harassment? This is an important question to ask, but most previous studies restricted their focus to the microlevel only. By contrast, few if any studies examined the possible macrolevel antecedents driving harassment-related worries among immigrants. This study aims to help fill this gap. Focusing on a 19-years period from 1986 to 2004 in Germany, we apply multilevel regression modeling techniques to repeated cross-sectional survey data collected among immigrants of Greek, Italian, Spanish, Turkish, and (ex-) Yugoslavian origin, linked with contextual characteristics. Our central finding is that German citizens' anti-immigrant prejudice is the key driver of longitudinal differences in immigrants' harassment-related worries. This association holds net of rival variables, such as fluctuations in media attention to ethnic harassment, as well as across all immigrant groups under study. These results bring us one important step further toward a better understanding of interethnic relations between immigrants and host society members.

## Introduction

Negative attitudes and behaviors of host society members toward immigrants continue to attract an immense amount of scholarly attention. Consequently, social science knowledge regarding the description and explanation of host-society members' ethnic harassment has become substantial (Semyonov et al., 2006; Zick et al., 2008; Ceobanu and Escandell, 2010; Hainmueller and Hopkins, 2014). Curiously, this body of work is not balanced by research on the consequences of anti-immigrant reactions for immigrants themselves. It is particularly unfortunate that no study seems to have explored the nexus between the prevalence of ethnic harassment and immigrants' concerns that the host society is biased against them. However, several reasons exist as to why such concerns—for brevity, henceforth dubbed “harassment- related worries”—deserve enhanced research attention. To illustrate, one can easily imagine that harassment-related worries impact negatively on immigrants' subjective well-being (Beier and Kroneberg, 2013), their acculturation attitudes (Christ et al., 2013), or their identification with the host society (Reeskens and Wright, 2014). Accordingly, harassment- related worries plausibly represent a considerable obstacle to immigrants' successful social integration. Beyond such applied relevance, investigating harassment-related worries in immigrants is important to resolve provoking theoretical puzzles. Extant research consistently finds that some minority group members tend to systematically underestimate their exposition to hostile practices emanating from majority members, whereas others are inclined to overestimate the occurrence of such intimidating acts (Major and Sawyer, 2009). Combined, both tendencies might be taken to imply that minority members' evaluations of an event as harassment on ethnic grounds occur relatively independently of the characteristics of the event itself but are mainly driven by personal characteristics. As applied to this study, this begs an intriguing question: Could it be that immigrants' harassment-related worries occur relatively independently of features of the macrolevel social contexts within which interethnic relations take place? Or does a comprehensive understanding of harassment- related worries necessitate accounting for such contextual characteristics? Providing adequate answers to this question is complicated by the fact that extant related work in this filed tends to focus on microlevel factors as antecedents of immigrants' perceptions of hostile intergroup behavior (Major and Sawyer, 2009; Dustmann et al., 2011; ten Teije et al., 2013; McGinnity and Gijsberts, 2016; Simonsen, 2016; Schaeffer, 2019; Steinmann, 2019). While this line of research doubtlessly uncovered several important insights, we know only little about the role that macrolevel factors play in shaping immigrants' beliefs that the host society is biased against them. Simonsen (2016), however, provides evidence that cross-national differences in majority members' anti-immigrant prejudice are associated with a greater likelihood that immigrants perceived their group to be discriminated against. Building on and extending this theoretical vantage point, the present study focuses on two factors that might shape immigrants' harassment- related worries: (a) majority members' anti-immigrant prejudice, and (b) mass media coverage of ethnic harassment. Empirically, we take advantage of a unique longitudinal data set containing information on the prevalence of harassment-related worries in immigrants of Greek, Italian, Spanish, Turkish, and (ex-)Yugoslavian origin living in Germany, covering the 1986 to 2004 period. This empirical source combines 20 waves of individual data (*n* = 32,744) with longitudinal statistics on native Germans' anti-immigrant prejudice and information from content analysis of newspaper reports. To the best of our knowledge, research covering such an extensive time frame and multiple groups of immigrants has not been available up to now. Beyond that, as we describe in detail below, the period under study shows considerable variation regarding the intensity of conflict between host society members and immigrants. This makes Germany an instructive test case to examine the nexus between contextual-level characteristics changing over time and individual-level harassment- related worries in immigrants, thereby complementing the insights from previous cross-national work (Simonsen, 2016).

## Theoretical Framework

### Conceptualizing Harassment-Related Worries Among Immigrants

Before we outline our theoretical expectations, we begin by clarifying the object of our inquiry. Building on general definitions of worrying (Borkovec et al., 1998; Gladstone and Parker, 2003), we define immigrants' worries concerning ethnic harassment as repetitive cognitive activities referring to feared future incidences of harassment on the grounds of their ascribed ethnic group membership. The central implication of this account is that immigrants' harassment-related worries do not need to be based on their factual experiences of intimidating or derogating acts nor on any “objective” likelihood of becoming a target of ethnic harassment. Instead, this definition puts immigrants' subjective appraisal of host society members' harmful intergroup conduct or, synonymously, ethnic harassment, center stage. This ethnic harassment occurs when host society members act with negative intent out of dislike for immigrants due to their believed ethnic group membership^1^. On an empirical level, acts of ethnic harassment might range from relatively frequent and mundane manifestations such as verbal or non-verbal derogation (see also Hayward et al., 2017, p. 351) to more extreme and rare forms, such as murder and physical violence (Allport, 1954; Virdee, 1995). It is important to keep in mind that harassment-related worries of immigrants are conceptually similar to but different from two longstanding neighboring constructs: (a) perceived ethnic discrimination and (b) intergroup anxiety. Simonsen (2016), e.g., conceives of perceived ethnic discrimination as “the subjective experience that one is treated unfairly because of one's group membership” (p. 375). The scope of the broad and diverse literature on perceived ethnic discrimination, however, typically does not cover more extreme forms of ethnic harassment such as anti-immigrant riots or physical violence, as we do. Further, most previous studies on perceptions of ethnic discrimination focus on the subjectively perceived prevalence of discriminatory activities. Deviating from this approach, the data at our disposal enable us to assess whether ethnic harassment is associated with worrying—a specific cognitive reaction.

Relatedly, intergroup anxiety—that is, “a feeling of worry, unease, or concern created by encounters or even thoughts of encounters with a member or members of a different social group (Stephan and Stephan 1985, 2017, p. 1),” also resembles the phenomenon we conceive of immigrants' harassment-related worries. However, intergroup anxiety and harassment-related worries differ with regard to the role played by personal encounters. For intergroup anxiety to occur, such encounters represent a necessary condition (see Stephan and Stephan, 1985)—but not for harassment-related worries. In fact, as we outline below, receiving information about the denigration of fellow group members might suffice to evoke such harassment-related worries. To approach the question as to what macrolevel factors shape immigrants' harassment-related worries, we employ a group threat framework as our theoretical perspective (Stephan et al., 2009). Two arguments support this perspective: First, harassment-related worries and threat perceptions show strong conceptual overlap, because they both represent cognitive appraisals of negative consequences attributed to outgroup members. Second, existing research documents that ethnic threat perceptions are susceptible to features of the social contexts within which immigrants and natives interact (e.g., Scheepers et al., 2002; Schlueter et al., 2013). Notice that for the present purposes, immigrants represent the ingroup, whereas host-society members—the source of potential threats to immigrants and harassment-related worries—constitute the outgroup. It seems promising to expect that immigrants' harassment-related worries will also be affected by macrolevel factors^2^. Below, we focus on two such factors: (a) majority members' anti-immigrant prejudice, and (b) mass media attention to ethnic harassment.

### Anti-immigrant Prejudice

Several perspectives suggest that anti-immigrant prejudice, broadly defined here as negative evaluations of immigrants based on their ethnic group membership (see Crandall and Eshleman, 2003) will heighten immigrants' worries concerning ethnic harassment.

For example, there is unequivocal evidence that prejudice represents a robust predictor of routine forms of ethnic harassment, such as derogatory comments, gestures, and behaviors in everyday interethnic encounters (Schütz and Six, 1996; Kauff et al., 2013).

Presumably, prejudiced communications and interactions also underlie the systematic discrimination of immigrants observed in the housing market (Klink and Wagner, 1999; Barwick and Blokland, 2015; Schlueter et al., 2018) and in the job market (Kaas and Manger, 2010). Besides individual exposure to discriminatory activities on the part of majority group members, peer communication about experiences of ethnic harassment constitutes a further plausible channel via which prejudice influences immigrants' harassment-related worries. Existing research also holds that anti-immigrant prejudice signals the social norms leading to manifest violence against immigrants (Dustmann and Preston, 2007). Ohlemacher (1994), for example, finds that a heightened negative public opinion climate precedes manifest violence targeted against migrants and refugees living Germany.

Thus, prejudice appears to affect immigrants' worries that the host society is biased against them across different domains. At first sight, this straightforward line of reasoning might lead one to think that there is nothing to question that prejudice has a positive impact on immigrants' harassment related worries. However, as mentioned in the introduction, solid theoretical and empirical arguments exist that speak against the view that anti-minority prejudice is a key driver of minority members' concerns about an anti-minority bias. On the one hand, minority group members have been found to underestimate the extent of being confronted with prejudice, a relation that has been attributed to self-presentational concerns or the motivation to avoid being stigmatized as a victim. On the other hand, prior research also shows that some minority group members become vigilant with regard to prejudice and discrimination, possibly in order to protect their self-esteem against disadvantage (Major and Sawyer, 2009). These tendencies might easily undermine the presumed impact of anti- immigrant prejudice on immigrants' perceptions thereof, which underlines the need for reinforced research efforts in this field.

*Hypothesis 1*: We expect that the likelihood that immigrants experience worries about ethnic harassment will be greater in periods characterized by higher levels of anti- immigrant prejudice than in periods characterized by lower levels of anti-immigrant prejudice.

### Mass Media Attention to Ethnic Harassment

Alone in the two decades under study, anti-immigrant violent acts in Germany caused the death of at least 100 immigrants (Die Zeit, 2015) and left many more injured. It is well-known that such extreme forms of ethnic harassment are regularly covered by the mass media and thus brought to a wide audience (Brosius and Esser, 1995), thereby enhancing the likelihood that large parts of the immigrant population become aware of the occurrence of ethnic harassment. Given that news reports of ethnic harassment often emphasize the role of immigrants as victims of host society members' negative behaviors mass media coverage of ethnic harassment might represent a further contributor to immigrants' harassment-related worries ^3^. Underlying this expectation is the basic notion that information transmitted by the mass media contributes to peoples' intergroup attitudes and behaviors, and that such influences increase with greater media attention, i.e., more frequent mass media reports on a given topic (Boomgaarden and Vliegenthart, 2009; Schlueter and Davidov, 2013; Schemer, 2014). Notice that in addition to immigrants' direct personal exposure to news on ethnic harassment harassment-related worries occur might also be shaped through indirect mass media experiences, e.g., via peer communication about news reports on ethnic harassment. Combined, this leads us to expect that greater mass media attention to ethnic harassment will increase immigrants' harassment-related worries.

*Hypothesis 2*: We expect that the likelihood that immigrants experience worries about ethnic harassment will be greater in periods characterized by a larger number of news reports on ethnic harassment than in periods characterized by a smaller number of news reports on ethnic harassment.

Before we examine the empirical merits of our theoretical expectations, we briefly consider the setting of our study – Germany, from 1986 to 2004.

## Research Setting: Immigrants in Germany, 1986 to 2004

As in other European destination countries, large parts of the immigrant population living in Germany originated from 1960s labor migration, with later admissions of family members further increasing the number of immigrants (Thränhardt, 1992). In 1986, the total number of immigrants was 4.6 million people (about 6 percent of the population). In the early 1990s a substantial number of refugees arrived in Germany and continued to increase the total number of immigrants. In 2004, the last year of our observational period, the total number of foreigners living in Germany was 6.7 million people, or 8.1% of the total population (Destatis, 2015). It is well-known that German host society members often react negatively to the arrival and presence of immigrants. For example, negative attitudes toward immigrants continue to represent a widespread social problem in Germany (Coenders and Scheepers, 2008; Schlueter et al., 2008). A similar conclusion follows with regard to the violent outbursts against immigrants and refugees noted above. These particularly severe forms of ethnic harassment reached a peak in the early 1990s (Ohlemacher, 1994), but they still occur on a regular basis [BMI (Bundesministerium des Inneren), 2014]. Given these fluctuations in interethnic conflict over time, Germany is an ideal case to examine if and to what extent anti-immigrant prejudice and news reports on ethnic harassment affect immigrants' harassment-related worries.

## Data and Measures

### Data

To examine our theoretical predictions we linked individual-level data from 20 waves of repeated cross-sectional surveys with contextual-level characteristics varying over time. Data for the “Ausländer in Deutschland” survey series were collected by the Marplan research institute (Marplan Forschungsgesellschaft, 1986-2004) using face-to-face interviews. For each of the five immigrant groups (immigrants of Italian, Spanish, (ex-)Yugoslavian, Greek, or Turkish origin), every wave comprised representative quota samples of ~*n* = 500 immigrants living in the West German federal states and Berlin. To be able to match our central contextual-level independent variable with these data, we focus here on data from the period 1986–2004. From 1986 to 1998 as well as in 2003 and 2004, immigrants were surveyed on an annual basis. From 1999 to 2002, the surveys were conducted biannually. In total, the pooled data set comprises *n* = 32,744 immigrants nested in 20 waves of repeated cross-sectional surveys. To our knowledge, this broad empirical source represents the longest time series of repeated cross-sectional surveys conducted among immigrants in Europe.

### Dependent Variable

#### Harassment-Related Worries

To assess immigrants' harassment-related worries, we take advantage of a single indicator that is available in the same format across all of the Marplan survey waves. Respondents were asked in the questionnaire to indicate if they feel worried (= 1) or do not feel worried (= 0) with regard to ethnic harassment (*Ausländerfeindlichkeit*). In asking respondents for their self-reported worries, this single indicator provides a global, well-suited assessment of immigrants' evaluation regarding the likelihood of seeing themselves or their fellow group members as targets of ethnic harassment.

### Independent Variables

#### Anti-immigrant Prejudice

We aggregate individual data from the *Politbarometer* survey series (Forschungsgruppe Wahlen, 2015) to operationalize German citizens' anti-immigrant prejudice as a characteristic of survey waves. The *Politbarometer* is a monthly poll based on probability sampling techniques, conducted among the German non-institutionalized general population aged 16 years and older. In the surveys, participants were asked in an open-ended question format: “What in your opinion is currently the most important problem in Germany?” and “And what is the second most important problem in Germany?” We averaged the percentages of respondents that indicated “foreigners” and/or “asylum seekers” as the most important or the second most important problem to form a proxy-measure of contextual-level anti-immigrant prejudice. Since the Politbarometer provides monthly survey data, we are able to construct this measure using a 3-months lag by considering the actual interview dates of the “Ausländer in Deutschland” surveys. For example, our measure for 1999 is based on the proportion of individuals mentioning immigrant-related keywords when answering the most-important-problem questions in January through March of 1999 as the survey was administered in March. In utilizing this measure, we follow several existing studies that demonstrate that responses to the most-important-problem question represent a valid indicator of negative attitudes toward immigrants (Ohlemacher, 1994; Lubbers and Scheepers, 2001; Boomgaarden and Vliegenthart, 2009)^4^.

#### Mass Media Attention to Ethnic Harassment

To operationalize mass media attention to ethnic harassment, we conducted a computer- assisted frequency analysis (Krippendorf, 2003) of the digitally available content of the conservative broadsheet *Frankfurter Allgemeine Zeitung* and the left-wing broadsheet *Die Tageszeitung* for the 1986–2004 period. We identified relevant articles by searching for key words such as “ethnic harassment” (*Ausländerfeindlichkeit*) or “ethnic hate” (*Ausländerhass*) in the headlines (Althaus et al., 2001) of all articles appearing up to 12 months before the start of the fieldphase of the surveys assessing immigrants' harassment-related concerns.

Irrespective of the different political learnings of both national newspapers, the trends in the attention paid to ethnic harassment in each news outlet were very similar (*r* = 0.92, *p* < 0.001). This indicates a considerable level of similarity in media attention to ethnic harassment across both news outlets, which reduces the risk of selection bias (Barranco and Wisler, 1999). We therefore averaged the number of articles from both newspapers to indicate media attention to ethnic harassment.

Note that anti-immigrant prejudice and mass media attention to ethnic harassment are strongly correlated (*r* = 0.76) but VIF values for both values are well below common cut-off points with 2.1 for both measures.

### Control Variables

Differences in the composition of the immigrant population over time (Kalter and Granato, 2002) might alter the prevalence of harassment-related concerns among immigrants. To reduce this possibility, we included several individual control variables in our models (see André et al., 2009; ten Teije et al., 2013). To assess immigrants' *ethnic group membership*, we employ five dummy variables to indicate whether the respondents were of (ex-)Yugoslavian, Greek, Italian, Spanish, or Turkish origin. *Sex* was coded with males as the reference category (1 = “female”). Respondents' *age* was originally measured in years. We recoded this variable in five categories 1 = “18–29 years”; 2 = “30–49 years”; 3 = “50–64 years”; 4 = “65 years and older.” Further, we classified respondents' *immigrant generation* according to whether they were born in Germany or not (born outside Germany = “first generation,” born in Germany = “second generation”). We assessed immigrants' employment status using a trichotomous variable (0 = “not in the labor force”; 1 = “unemployed”; 2 = “working”).

*Educational attainment* was assessed with years of fulltime formal education. We recoded these scores according to the ISCED-Scheme (UNESCO Institute for Statistics, 2012) in four categories: <7 years of education = “ISCED 0–1”; 7–11 years of education = “ISCED 2”; 12–13 years of education = “ISCED 3”; more than 13 years of education = “ISCED 4–6.” Survey participants were also asked to evaluate *Germans' attitude toward foreign co-workers at the workplace*^5^. Answer options were given on a scale from 1 (“very friendly”) to 6 (“very unfriendly”). We employ this item to account for interindividual differences in respondents' preexisting sentiment toward Germans and the German host-society. Finally, we also control for differences in respondents' *German language proficiency*. The interviewers evaluated immigrants at the end of the survey questionnaires on both their reading and speaking skills in German. Reading skills were assessed using a four-point scale with the endpoints 1 = “has perfect skills in reading German” and 4 = “is unable to speak German.” Speaking skills were evaluated using a five-point scale from 1 = “speaks German perfectly” to 5 = “no verbal communication in German possible.” As the two language variables were strongly correlated (*r* = 0.81, *p* < 0.001), we averaged them to form a single indicator. Subsequently, we reversed the coding so that higher values indicate higher German language skills. Descriptive statistics are reported in Table 1. Moreover, we relied on list-wise deletion to deal with missing information^6^.

**Table 1:** Descriptive statistics of dependent and independent variables (*n* = 32,744; *N* = 20).

	**Range**	**Mean**	**SD**
**Dependent variable**			
Worried about ethnic harassment	0–1	0.19	
**Independent variables**			
Spaniards	0–1	0.19	
Italians	0–1	0.20	
(Ex)Yugoslavians	0–1	0.20	
Greeks	0–1	0.20	
Turks	0–1	0.21	
ISCED 0–1	0–1	0.16	
ISCED 2	0–1	0.73	
ISCED 3	0–1	0.09	
ISCED 4–6	0–1	0.02	
Age: 18–29	0–1	0.30	
Age: 30–49	0–1	0.49	
Age: 50–64	0–1	0.19	
Age: > 64	0–1	0.02	
Female	0–1	0.43	
First generation	0–1	0.81	
Second generation	0–1	0.19	
Not in the labor force	0–1	0.29	
Unemployed	0–1	0.07	
Employed	0–1	0.64	
German attitudes at the workplace	1–6	2.61	0.96
German language proficiency	1–5	2.01	0.67
Immigrants/refugees currently most important problem	0–0.46	0.12	0.11
Media attention to ethnic harassment	3–135	21.19	28.51

## Results

### Descriptive Results

We begin presenting our results by taking a descriptive look at the trajectory of immigrants' harassment-related worries. These observations are based on aggregated scores only, but they provide an informative empirical vantage point for the subsequent multivariate multilevel regression analyses. Preliminary analyses indicated that the group-specific scores of derogation-related concerns for immigrants of Greek, Italian, Spanish, Turkish, and (ex-)Yugoslavian origin were quite similar. To simplify matters, we averaged the group-specific trajectories and focus here on the overall development of immigrants' harassment-related worries only.

**Figure 1:**
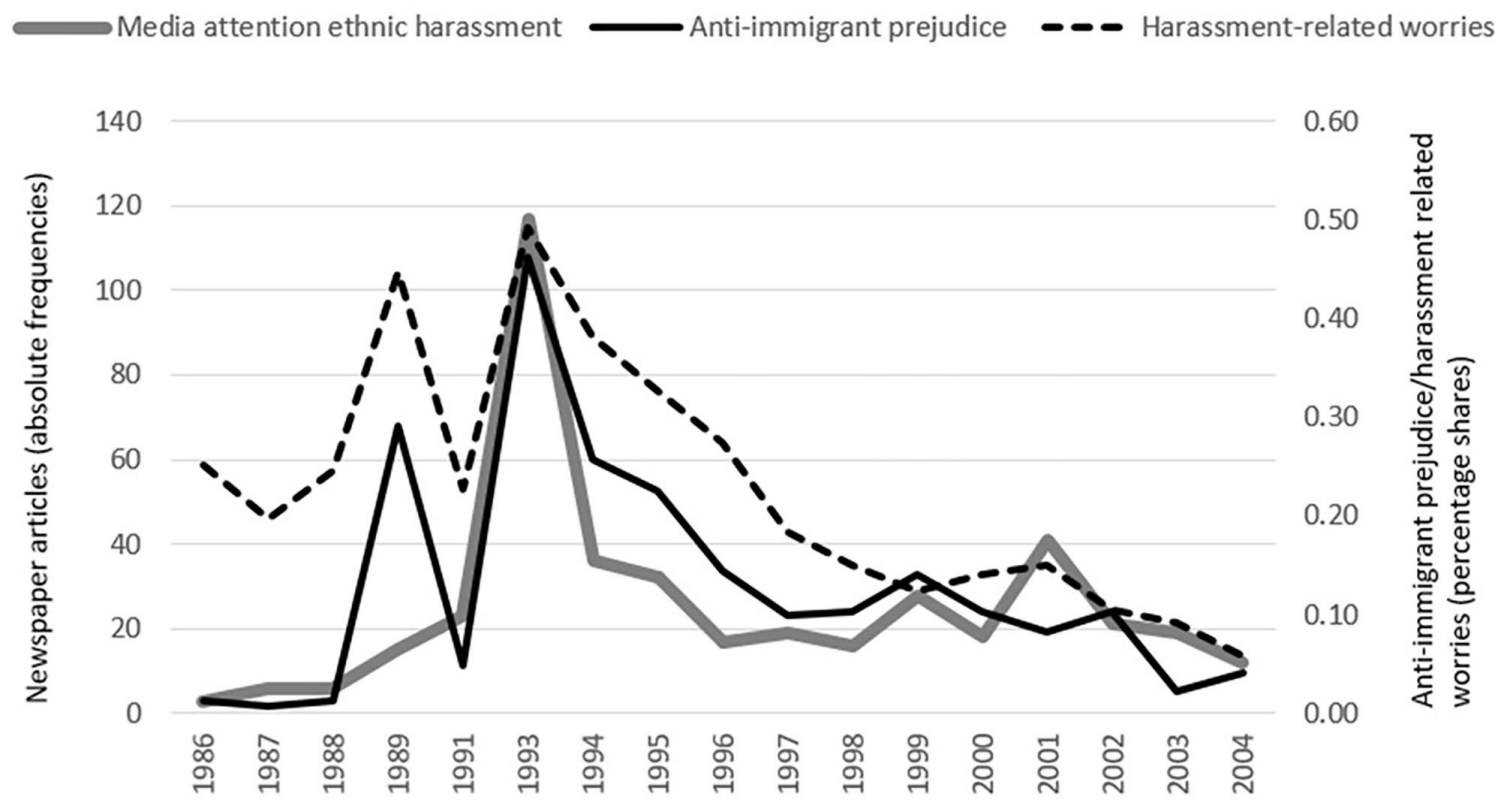
Trajectories of immigrants' derogation-related concerns and majority members' anti-immigrant prejudice in Germany, 1986–2004.

Figure 1 suggests that immigrants' harassment-related worries (dotted line), majority members' anti-immigrant prejudice (straight black line), and mass media attention to ethnic harassment (bold gray line) display quite similar trajectories over time. Starting with relatively low scores at the beginning of the observational window in 1986, both harassment- related worries and anti-immigrant prejudice show a sharp rise in 1989—the year of the electoral breakthrough of the German radical rightwing party *Die Republikaner* (Mudde, 2003)—and decline to their initial level thereafter. Anti-immigrant prejudice, harassment- related worries and mass media attention to ethnic harassment reach their maxima in 1993, after a period well-known for its exceptional rise in widespread anti-immigrant violent acts (Ohlemacher, 1994). For the remaining time, the data reveal a gradual decrease for each of the three variables up until the end of the observational window in 2004. This common trend was interrupted only by smaller peaks in anti-immigrant prejudice in 1999 as well as in harassment-related worries and mass media attention to ethnic harassment in 2000, respectively. From a broader perspective, then, these descriptive findings point to an overall decrease in immigrants' harassment-related worries, paralleled by similar developments in anti-immigrant prejudice and mass media coverage of ethnic harassment. Yet irrespective of this general trend, the data also shown remarkable common spikes which point to a positive relation of prejudice, respectively, mass media coverage with immigrants' harassment-related worries. However, we do not know whether this suggestive evidence remains intact once we submit the data to a more systematic empirical test. To achieve better insights on this issue, we now turn to the results from hypothesis testing using multilevel modeling techniques.

### Results From Hypothesis Testing

We begin by noting that in our repeated cross-sectional survey data, individual immigrants (level 1) are nested in surveys (level 2). To deal with this data structure adequately, we employed two-level hierarchical regression models with a logit link (Hox, 2010). These models account for the clustering of respondents within surveys by treating each survey wave as a separate context and specifying a variance component that allows the intercept (i.e., the proportion voicing ethnic harassment-related worries) to vary across surveys. All models are based on penalized quasi-likelihood estimation procedures. Table 2 shows that we started with an “empty” model (model 1) that contains no covariates but where the intercept varies randomly between contexts. Converting the results into a variance partition coefficient indicated that ~13% [0.495/(π^2^/3) + 0.495] of the total variance in immigrants' harassment-related worries were situated between years^7^. Consistent with the descriptive results above, this result indicates a substantial amount of contextual-level, longitudinal differences in the dichotomous dependent variable.

**Table 2:** Logistic multilevel model predicting harassment-related worries (*n* = 32,744, *N* = 20).

	**Model 1**	**Model 2**	**Model 3**	**Model 4**
Intercept	−1.480* (0.151)	−4.218* (0.206)	−4.632* (0.243)	−4.842* (0.199)
**Individual level variables**				
Spaniards (Ref.)		-	-	-
Italians		−0.245* (0.055)	−0.245* (0.055)	−0.245* (0.055)
(Ex-)Yugoslavians		0.644* (0.049)	0.644* (0.049)	0.644* (0.049)
Greeks		0.098 (0.052)	0.098 (0.052)	0.098 (0.052)
Turks		0.311* (0.050)	0.311* (0.050)	0.311* (0.050)
ISCED 0–1 (Ref.)		-	-	-
ISCED 2		0.237* (0.047)	0.236* (0.047)	0.236* (0.047)
ISCED 3		0.270* (0.074)	0.300* (0.074)	0.299* (0.074)
ISCED 4–6		0.494* (0.115)	0.493* (0.115)	0.494* (0.115)
Age: 18–29 (Ref.)		-	-	-
Age: 30–49		0.023 (0.040)	0.022 (0.040)	0.022 (0.040)
Age: 50–64		−0.057 (0.052)	−0.057 (0.052)	−0.058 (0.052)
Age: > 64		−0.004 (0.131)	−0.006 (0.131)	−0.009 (0.131)
Female		0.059 (0.036)	0.058 (0.036)	0.058 (0.036)
First generation (Ref.)	-	-	-	-
Second generation		−0.096* (0.046)	−0.096* (0.047)	−0.097* (0.049)
Not in labor force (Ref.)	-	-	-	-
Unemployed		−0.198* (0.066)	−0.198* (0.066)	−0.198* (0.066)
Employed		−0.070 (0.041)	−0.070 (0.041)	−0.070 (0.041)
German attitudes at the workplace		0.750* (0.017)	0.750* (0.017)	0.751* (0.017)
German language proficiency		0.048 (0.027)	0.048 (0.027)	0.047 (0.027)
**Contextual-level variables**				
Media attention to ethnic harassment			0.016* (0.006)	−0.006 (0.006)
Immigrants/refugees currently most important problem				6.097* (1.261)
**Random effect**				
Var(year)	0.471	0.465	0.336	0.152

*= p < 0.05.

Model 2 builds on and extends the “empty” model by adding the individual-level control variables. The main aim here was to account for compositional differences among immigrants for the period under study, which otherwise might distort the subsequent results.

In short, the results show that the estimate of the random effect from model 1 to model 2 remains virtually unchanged. This means that compositional differences explain very little of the longitudinal differences in immigrants' derogation-related worries. The specific findings for the control variables are only considered briefly here, as they are not the main focus of our research. The data reveal that relative to Spaniards, all but Italian respondents were more likely to voice concerns about ethnic harassment. Accordingly, (ex-)Yugoslavians had the highest odds, followed by Turks and Greeks^8^. The results also provide evidence that more educated immigrants were more likely to exhibit harassment-related worries. Migrants with medium levels of education (ISCED 2-3) were between 27 and 35% (e^.237^–1 and e^.297^–1) more likely to mention ethnic harassment-related concerns than migrants with a low level of education (ISCED 0–1). This figure is even higher for immigrants with a high level of education (~64%). However, only very few migrants in our data reported ISCED levels >3 (ca. 2%, see Table 1). A further corollary finding is that immigrants who evaluate Germans' attitude toward foreign co-workers at the workplace as relatively more negative are more likely to voice worries about ethnic harassment. With regard to the remaining control variables, our findings reveal little difference with respect to gender or age. In addition, members of the second generation as well as unemployed members of the labor force were less likely to be worried about ethnic harassment. The subsequent models shift attention to the contextual-level independent variables and are key in answering our research question. Model 3 adds the measure of mass media attention to ethnic harassment. Providing preliminary support for the idea that more intense mass media attention heightens immigrants' harassment-related worries, the data reveal a significantly positive parameter estimate (b = 0.016). Model 4 extends the analyses by including the indicator assessing anti- immigrant prejudice. In this model, the relation between mass media attention of ethnic harassment and harassment-related worries observed before disappeared. Probably due to the strong co-variation with anti-immigrant prejudice over time, the parameter estimate for the mass media indicator changes its sign and is no longer distinguishable from zero. The central finding from model 4 is that greater anti-immigrant prejudice leads to a remarkable increase in the odds of immigrants experiencing harassment-related worries. To illustrate, the data show that the odds of immigrants reporting harassment-related worries more than double (e^.10*6.463^–1 = 91%) for survey waves where anti-immigrant prejudice is 10% points (~ one standard deviation) above its mean. Further, the contextual variance situated between survey waves decreases from 0.336 (model 3) to 0.123. This indicates that German citizens' anti-immigrant prejudice accounts for 63% of the residual longitudinal variance in immigrants' harassment-related worries—a large effect. To further probe the robustness of the results, we estimated model 4 for each of the five immigrant groups separately (not shown in the table). These supplementary analyses revealed that Spaniards' odds of voicing concerns about ethnic harassment were the most sensitive to changes in the majority populations anti-immigrant prejudice (+118 percent), followed by Italians (+99%), (ex-)Yugoslavians (+93%), Greeks (+87%), and Turks (+70%). Even though Turks were the least sensitive immigrants to majority members' anti- immigrant prejudice, the magnitude of this relation was still comparable to the difference between migrants with the lowest and highest education levels. In conclusion, we find unequivocal evidence for *hypothesis 1* according to which stronger anti-immigrants prejudice increases harassment-related worries in immigrants. However, the assumption that mass media attention to ethnic harassment heightens harassment-related worries as stated in *hypothesis 2* receives no consistent empirical support^9^.

## Conclusion

This study sought to shift scholarly attention to the role of contextual-level sources for shaping immigrants' harassment-related worries. Drawing upon repeated cross-sectional survey data from immigrants living in Germany spanning the period from 1986 to 2004, this study is the first that approaches this task from a longitudinal perspective. Controlling for a range of individual-level characteristics, the results provide evidence that differences in immigrants' harassment-related worries over time are centrally shaped by fluctuations in majority members' anti-immigrant prejudice. This is a novel finding, with important implications for theory and research on the social integration of immigrants. First, from a more general perspective, it is noteworthy that the present results are consistent with much prior social science inquiry underlining the need to account for contextual characteristics to better understand differences in immigrants' integration into host societies. Another implication of the current results is that worries among immigrants that the host-society is biased against them cannot be attributed to personal characteristics alone. Instead, by adding the insight that harassment-related worries partly represent a response to host-society members' prejudice, the present findings underline the need to account for the interdependency of ethnic relations between host-society members and immigrants.

This conclusion should also be consequential for policy makers and activists. Accordingly, those interested in successful interethnic relations among immigrants and host society members are well advised to take efforts to prevent or reduce the prevalence of anti- immigrant prejudice in host societies.

This study also has various limitations, many of which point to promising avenues for future research. For instance, although our research was based on an unusually broad empirical source, data limitations did not allow us to combine the current longitudinal research perspective with an examination of spatial differences in immigrants' hostility- related worries. Specifically, differences in hostility-related worries in immigrants across spatial contexts (e.g., municipalities) might plausibly be associated with spatial variation in host society members' anti-immigrant prejudice. Due to absent small-scale spatial information we also refrained from investigating the possible impact of local anti-immigrant events such as demonstrations, riots or other violent acts on immigrants' views that host society members are biased against them. Could it be that the spatial distance to the location of an anti-immigrant event is irrelevant for the strength of harassment-related worries? Or do harassment-related worries increase in response to local racist protests or acts of violence?

Data permitting, future research might productively explore the relevance of spatial contexts for different sources of harassment-related worries in immigrants.

Further insights might also be gained from differentiating between harassment-related worries related to one's ethnic ingroup as different from worries related to oneself.

For example, related research finds that minority members commonly perceive discriminatory activities targeted against their ingroup to occur more often as compared to personal experiences of discrimination (Major and Sawyer, 2009). Accordingly, future research might investigate whether a similar pattern of results also holds when distinguishing between harassment-related worries with regard to oneself as different from worries related primarily to one's ethnic ingroup.

Finally, as mentioned above, the current results do not support the assumption that immigrants' harassment-related beliefs are shaped by the amount of mass media attention to ethnic harassment. Initially, the data revealed a significantly positive parameter estimate for the indicator of mass media coverage on ethnic harassment. Yet once we extended our model to include majority members' anti-immigrant prejudice, the parameter estimate for the news reports variable became statistically insignificant and changed its sign. This result might be taken to indicate that mass media coverage on ethnic-harassment has little to add in shaping immigrants' harassment-related concerns. However, it is well-known that many aggregate characteristics tend to move together over time (Janoski and Isaac, 1994), which often implicates methodological difficulties. Presumably, the longitudinal co-variation between the average level of Germans' anti-immigrant prejudice and the frequency of newspaper articles on ethnic harassment represents no exception from this. Thus, more conclusive insights regarding the possible impact from mass media reports on ethnic harassment on subjectively experienced worries await additional empirical results using alternative research designs.

Further, it should also be acknowledged that our frequency analyses of news reports on anti-immigrant events was based on German-speaking broadsheets only. It is conceivable that content analyses of ethnic newspapers appearing in Germany (Halm, 2006) might deliver alternative results regarding the frequency of news reports on ethnic harassment.

These limitations and directions for future work notwithstanding, it is important to keep in mind this study's main contribution to the extant literature – namely, the novel finding that longitudinal differences in immigrants' harassment-related worries are systematically shaped by majority members' anti-immigrant prejudice.

## Data Availability Statement

The datasets generated for this study are available on request to the corresponding author.

## Ethics Statement

Ethical review and approval was not required for the study on human participants in accordance with the local legislation and institutional requirements. The patients/participants provided their written informed consent to participate in this study.

## Author Contributions

All authors listed have made a substantial, direct and intellectual contribution to the work, and approved it for publication.

## Conflict of Interest

The authors declare that the research was conducted in the absence of any commercial or financial relationships that could be construed as a potential conflict of interest.

## Footnotes

^1^Throughout this article, instead of the more often used term racial harassment we prefer the term ethnic harassment. Doing so helps to emphasize that criteria such as ancestry, language, or physical markers leading to ascriptions of group membership are historically contingent constructions and that their social significance does not rely on observable biological difference between people, as the term racial might imply.

^2^Studies relying on arguments derived from group threat theory typically also include measures of economic conditions (e.g., unemployment rates) as well as group size. However, their aim is usually to explain anti-immigrant attitudes of the majority population. In times of, for example, economic hardship, majority members might feel more threatened by immigrants thus increasing the prevalence of anti-immigrant attitudes. Here, we use anti-immigrant to explain why some immigrants are more likely to fear harassment by majority members. There are few reasons to suspect that economic conditions have an independent effect on this likelihood that is not transmitted via the effect of anti-immigrant attitudes. This is also borne out by sensitivity analyses including the German unemployment rate where the coefficients of anti-immigrant attitudes (b = 6.46, *p* < 0.001 vs. b = 5.14, *p* < 0.001) and media coverage (b = −0.00, *p* = 0.173 vs. b = −0.00, *p* = 0.756) remain essentially unchanged.

^3^We acknowledge that extant research also documents that news reports on immigrants and immigration often portray immigrants in negative ways. It might seem tempting to examine if and to what extent this negative news coverage of immigrants contributes to heightened harassment-related worries in immigrants. However, negative news coverage of immigrants are known to centrally shape host-society members' negative sentiments toward immigrants (Schlueter and Davidov, 2013), or anti-immigrant prejudice. This means that any influence from negative mass media portrayals of immigrants on their harassment-related worries should occur indirectly via host-society members' anti- immigrant attitudes, which we already include in our theoretical expectations.

^4^We examined the robustness of our findings using additional proxy-items to assess anti- immigrant negativity. To this end, we constructed a dummy-variable from the Politbarometer Survey series measuring respondents' voting intentions for radical rightwing parties (0 = other parties, 1 = *Die Republikaner*, DVU, NPD), given that anti- immigrant prejudice is a known predictor of such voting intentions. The results were highly comparable (i.e., a significant association of German citizens' average intention to vote radical rightwing parties covaries positively with immigrants' harassment-related worries) to our findings reported in the main analyses.

^5^This question was presented to all survey participants regardless of their occupational status.

^6^Replication code can be found at doi: 10.17605/OSF.IO/JN9ZH.

^7^The individual-level variance in logistic models is fixed to π^2^/3 (Hox, 2010).

^8^We note that Germany admitted a substantial number of (ex-)Yugoslavian refugees during the early 1990s, a time of comparatively high levels of anti-immigrant sentiment (see Figure 1). Perhaps (ex-)Yugoslavians had been targets of the high levels of anti- immigrant sentiment, which potentially explains their higher levels of ethnic harassment worries.

^9^In supplemental analyses, we explored the plausible idea suggested by a reviewer that highly educated immigrants tend to be especially sensitive to forms of discrimination and ethnic harassment. However, the cross-level interaction models we estimated to test this suggestion showed that the effect of anti-immigrant attitudes on immigrants' harassment-related worries does not vary with individual's educational attainment.
